# Artificial light at night intensifies effects of a parasitic flatworm on the water flea *Daphnia magna*

**DOI:** 10.1098/rsbl.2025.0373

**Published:** 2025-09-24

**Authors:** Nedim Tüzün, Franz Hölker, Luc De Meester

**Affiliations:** ^1^ Leibniz-Institute of Freshwater Ecology and Inland Fisheries (IGB), Berlin, Germany; ^2^ Institute of Biology, Free University of Berlin, Berlin, Germany; ^3^ Laboratory of Freshwater Ecology, Evolution and Conservation, KU Leuven, Leuven, Flanders, Belgium

**Keywords:** light at night, *Strongylostoma simplex*, parasite, microturbellarian, egg predation

## Abstract

Artificial light at night can strongly alter organismal traits, but its role in shaping species interactions remains poorly understood, especially so in aquatic ecosystems. By capitalizing on a recently discovered antagonistic interaction between a brood-parasitic flatworm and *Daphnia magna* water fleas, we tested whether this interaction depends on exposure to artificial light at night. During a 19 day laboratory population growth experiment, we manipulated flatworm presence and night-time light conditions in a full-factorial design. We confirmed the negative effects of flatworm predation on *Daphnia* abundance at the population level. Importantly, we showed that the flatworm-caused reduction in the final population size of *Daphnia* under artificial light at night was twice as strong (81%) compared to under dark–night conditions (39%). Our findings are relevant when it comes to assessing the impact of artificial light at night on the development of *Daphnia* populations and thus top-down control of phytoplankton. Freshwater ecosystems in urbanized areas, where this parasitic interaction was first encountered, may be especially at risk, as these are typically exposed to high levels of stress factors, including light pollution.

## 1. Introduction

Human-induced environmental change shapes not only organismal traits but also species interactions [1]. Artificial light at night (ALAN) is a pervasive stressor for a wide range of taxa, influencing life history, behaviour and physiology, which in turn influence species interactions and ultimately community structure and ecosystem functions [2–4]. Whether and how species interactions are shaped by ALAN has recently gained attention among ecologists [5]. Aquatic habitats are suggested to be particularly threatened by ALAN, because aquatic organisms often have reduced available refuge to avoid light exposure, especially so in urban ponds with limited structural complexity [6] and are expected to be exposed to high levels of ALAN [7]. Despite this, the effects of ALAN on species interactions in aquatic ecosystems remain understudied [7].

Natural temporal light patterns produced by nocturnal celestial bodies are important environmental cues for many animals, including aquatic species [8–10]. For example, moonlight can induce the vertical migration of zooplankton down to a depth of 100 m [11]. The high sensitivity of aquatic organisms to low-intensity natural light makes them susceptible to disturbance even by low-intensity ALAN [7]. It is known that ALAN interrupts natural diel vertical migration patterns [12–14], where zooplankton species stay in deeper water layers during the day to minimize the risk of predation by visual planktivorous fish and only ascend to the surface water layers at night to feed on phytoplankton and microzooplankton [15]. Disruptions of diel activity and habitat selection patterns can result in altered temporal niche partitioning, which in turn can influence species interactions, for instance resulting in increased predation rates when interactors occupy the same temporal niche [5]. Aside from changed activity patterns, ALAN may also alter species interactions by imposing physiological stress on the interacting organisms (e.g. [10,16]).

**Figure 1.**
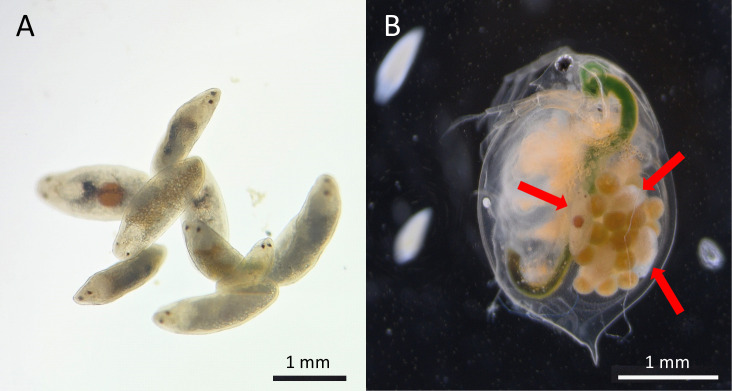
The typhloplanid flatworm *Strongylostoma simplex* (A) and the water flea *Daphnia magna* (B). Flatworms inside the brood chamber of a water flea with eggs are indicated with arrows. Note also on the left side of the water flea the two free-swimming flatworms. Photo credit for panel A: Mareike Brehm-Benedix.

To test whether ALAN has an influence on species interactions in aquatic ecosystems, we capitalized on a recently discovered interaction between a flatworm and *Daphnia magna* water fleas [17]. The water flea *Daphnia*, a key ecological interactor in freshwater ecosystems, is well suited for this question. First, *Daphnia* show a changed pattern of diel vertical migration under ALAN, with the animals staying closer to the bottom during both day and night [12,14]. Second, *Daphnia* have numerous antagonistic interactions with a wide range of organisms. We have recently shown that the typhloplanid flatworm *Strongylostoma simplex*, an egg parasite of *Daphnia* (figure 1), reduced survival and offspring production in *D. magna* [17]. Here, we first aim to test the hypothesis that the effects of the flatworm on *Daphnia* individuals translate into changes at the population level. We also aim to test the hypothesis that ALAN significantly influences the effects of flatworms on *Daphnia* abundance. While little is known about the diel vertical migration patterns of flatworms (e.g. [18]), we predict the low-depth preference of *Daphnia* under ALAN to result in an increased encounter rate between *Daphnia* and flatworms (assuming the flatworm is benthic: [19]). We therefore expected a reduced *Daphnia* abundance under ALAN in the presence of flatworms.

## 2. Material and methods

### (a) Experimental settings

To explore the effects of flatworms on *Daphnia* abundance under ALAN, we conducted a population growth experiment over 19 days. The experimental design consisted of a flatworm treatment (flatworm absent/present) crossed with an ALAN treatment (dark at night/light at night), using four replicates per treatment combination (*n* = 16). Treatments were assigned to experimental units using a randomization procedure.

To produce the light environment during day and night, we used LED lamps (Sylvania L300, 3000K, warm-white LED; Feilo Sylvania International Group, Hungary) and used neutral density filter foil (Reinan, USA) wrapped around the lamps to dim the light intensity for the ALAN treatment. Illuminance was measured on top of the jars with a sensitive illuminance metre (ILT-1600; International Light Technologies, USA). Both spectral distribution and correlated colour temperature (CCT) were measured using a spectroradiometer with a measurement range of 380−780 nm and 4.5 nm wavelength resolution (JETI Specbos 1211 UV; Jena Technische Instrumente, Jena, Germany; see also electronic supplementary material, figures S1 and S2). Illuminance and CCT during day hours were *ca* 400 lx (approximately 6.4 µmol photons m^−2^ s^−1^) and 3963 K, respectively. For the ALAN treatment, illuminance and CCT during night hours were *ca* 35 lx (approximately 0.5 µmol photons m^−2^ s^−1^) and 2978 K, respectively, compared to <0.02 lx (approximately 0.0004 µmol photons m^−2^ s^−1^, CCT below detection limit) for the control treatment. Both illuminance and CCT were within the typical range of recorded ALAN in urban areas, albeit the illuminance was at the higher end of that range [20].

Jars assigned to the flatworm treatment received five individual flatworms of similar size (*ca* 0.5 mm) at the beginning of the experiment. Flatworms were collected from a small artificial water body in a cemetery in Berlin and were identified as *Strongylostoma simplex simplex* [17]. These predatory freshwater flatworms have been previously recorded from natural lakes (see references in [17]) where *Daphnia* also occurs (e.g. Lake Mývatn in Iceland [21]). We used 1 l cylindrical glass jars (84 mm diameter, 210 mm height, WECK, Germany) filled with 750 ml of dechlorinated tap water. Each experimental population was started with seven *D. magna* individuals of mixed age: one adult, one subadult and five newly born juveniles. This starting composition was chosen to represent a realistic *Daphnia* population structure, as well as to reduce the possibility that all eggs would be attacked from the very start of the experiment, preventing any population development in the flatworm treatments, in case we were to start with mature adults only [17]. The number of *Daphnia* versus flatworms in experimental jars was informed by their relative abundance in the source habitats of the flatworms (N. Tüzün 2023, unpublished data). We used a single *D. magna* clone for this experiment, collected in 2022 from a very small artificial water pond in a cemetery in Berlin (52°31′19.6″ N 13°30′55.6″ E). The clone was kept in culture under standardized conditions (20°C, 14 : 10 light : dark photoperiod) in the laboratory of IGB Berlin (clone code: ZEN-4). The habitat from which the *Daphnia* clone was isolated did not contain the flatworm at the moment of isolation; therefore, we assume these *Daphnia* to be naive to the presence of *Strongylostoma simplex*. The experimental populations were fed daily with the green algae *Acutodesmus obliquus* at 1 mg C l^-1^. We refreshed the medium (dechlorinated tap water) three times per week.

The experiment was run in a temperature-controlled incubator (Pol-Eko ST3 Smart, Poland) at 20°C with a photoperiod of 14 : 10 light : dark (typical for August in Berlin). The experiment ran for 19 days, during which we manually counted the number of *Daphnia* when we refreshed the medium, i.e. three times per week. Dead individuals were removed from experimental jars. When counting the *Daphnia*, we also checked the number of worms in each jar and added new flatworms when necessary. The average number of flatworms per experimental jar throughout the trial period was 4.64 ± 0.64 (mean ± s.d.) for the control and 4.72 ± 0.57 for the light pollution treatment and was not significantly different between these groups (Wilcoxon test: *W* = 610, *p* = 0.577).

### (b) Statistical analyses

To test for the effects of flatworms on *Daphnia* abundance in the absence and presence of night-time light pollution, we constructed a linear model with time of the experiment (continuous), flatworm treatment (categorical: flatworms absent/present) and light pollution treatment (categorical: dark at night/light at night) as fixed effects. We included all interaction terms of these fixed effects in the model. Given the typical nonlinear shape of *Daphnia* abundance over time, we fitted a second-order polynomial function of time. In addition, we fitted logistic growth curves to test for differences in carrying capacity and growth rate during the exponential phase. These are reported as electronic supplementary material, table S1 and figure S3. Note that logistic growth models could only be estimated for the non-flatworm treatments, as the models would not converge for the flatworm treatment groups due to the fact that they did not reach a plateau. Pairwise comparisons of treatment effects and regression slopes using estimated marginal means were calculated using the R package *emmeans* [22]. Logistic growth models were fitted using the R package *nlme* [23], and the ‘reduced’ (no treatment differences in logistic curve parameters) versus ‘full’ (treatment-specific logistic curve parameters) models were compared using a likelihood ratio test. All analyses were performed in R v. 4.3.2 [24].

## 3. Results

**Figure 2.**
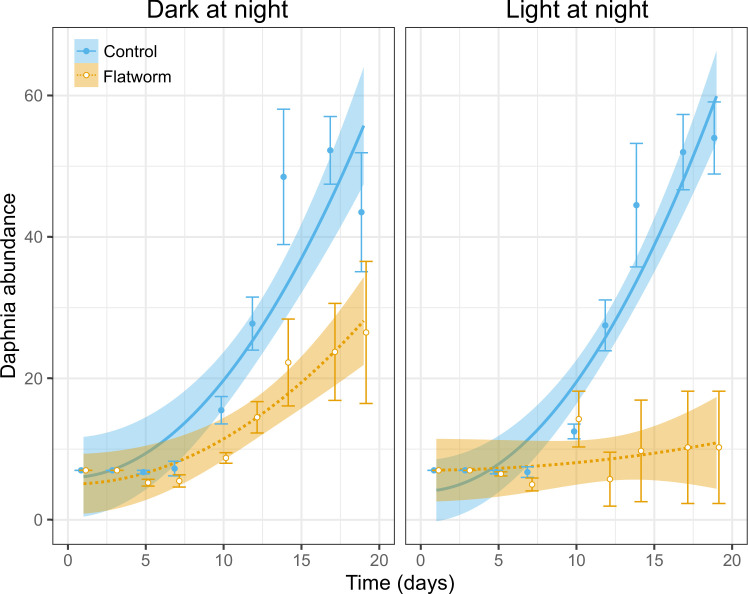
*Daphnia* abundance over time as a function of the presence or absence of predatory flatworms and the presence or absence of light pollution at night. The left panel represents the no light pollution (i.e. dark at night) condition, and the right panel represents the light pollution condition. Shown are regression lines for the control (blue, full line) and flatworm treatments (yellow, dashed line). Shown are also raw means (±1 s.e.) per time point. Bands around lines represent 95% confidence intervals (derived from the linear model; see table 1). Logistic growth curves fitted for control treatments are shown in electronic supplementary material, figure S3.

*Daphnia* abundance on average increased over time, with a steeper increase after the relatively stable first week (indicated by the significant quadratic term of time, table 1, figure 2). The increase in *Daphnia* abundance over time was on average less pronounced in the flatworm treatment compared to the no-flatworm treatment, and this pattern was more prominent after the first week of the experiment (time^2^ × flatworm treatment, table 1, figure 2). The significant three-way interaction further shows that in the light pollution treatment, the flatworm-induced negative effect on the increase in *Daphnia* abundance over time was more pronounced compared to the control light conditions, especially towards the end of the experiment (table 1, figure 2). This is further reflected as a significant difference between the linear growth rates (i.e. slopes) of flatworm-exposed *Daphnia* at control versus light pollution conditions (contrast test: *t* ratio = 2.59, *p* = 0.011). Considering only the no-flatworm treatments, the logistic growth curves did not differ between the control and light pollution treatments (likelihood ratio test = 1.71, *p* = 0.634, electronic supplementary material, figure S3); this is reflected in the similar values for the three logistic curve parameters across light pollution treatments (electronic supplementary material, table S1).

**Table 1.** Result of the linear model testing for effects of time, flatworm treatment, light pollution treatment, as well as their interactions, on *Daphnia* abundance. Note that time was included as a second-order polynomial term (implemented using the *poly(x,2*) function in R).

fixed effects	d.f.	*F*-value	*p*-value
time^2^	2	97.44	**<0.001**
flatworm treatment	1	67.83	**<0.001**
light pollution treatment	1	2.15	0.145
time^2^ × flatworm	2	34.41	**<0.001**
time^2^ × light pollution	2	1.04	0.357
flatworm × light pollution	1	2.69	0.103
time^2^ × flatworm × light pollution	2	3.97	**0.021**

Significant *p*-values (*p* < 0.05) are indicated in bold.

## 4. Discussion

Our results show that ALAN can exacerbate the adverse effects of brood-parasitic flatworm on *D. magna* water fleas. The interaction between these *Strongylostoma* and *Daphnia* was recently described for the first time, with observations clearly suggesting an egg parasitism behaviour of the flatworm, with first indications that survival (possibly due to injury during infection) and offspring production of *D. magna* can be strongly reduced in the presence of flatworms [17]. In the current study, we indeed confirm that this impact has negative effects on population development. Importantly, we show that the reduction in *Daphnia* abundance caused by flatworm parasitism interacts synergistically with ALAN, i.e. it is twice as pronounced under ALAN when compared to control light conditions (final population size reduced by 81% versus 39%, respectively). Our findings have relevance for assessing the impact of ALAN on the development of *Daphnia* populations and thus top-down control of phytoplankton in standing freshwater ecosystems, especially in urban areas, as these are typically exposed to high levels of light pollution [7].

While organismal responses to ALAN are well documented [3,4], there have been far fewer studies studies on species interactions under ALAN [5,25]. Light pollution can shape species interactions among others by altering behaviour and physiology in a way that affects the encounter rate between the interactors, as illustrated for host–parasite interactions in aquatic systems [26]. Fish predation, which also induces zooplankton to reside at greater depths in the water column, has been linked to increased parasitic infection in *Daphnia* due to increased exposure to parasite spores, which are found in the sediment [27]. Most typhloplanid flatworms prefer benthic habitats [19], which may explain our observation that the flatworms have a stronger impact under ALAN. Nevertheless, as we have not explicitly tested for altered depth preference in *Daphnia* under ALAN in the current study, this idea remains to be experimentally verified. An alternative pathway is that ALAN-induced physiological impacts (e.g. in phytoplankton [28]; in *Daphnia* [29]; in fish [30]) may have an effect on resource allocation, potentially making *Daphnia* more vulnerable to flatworm parasitism.

Increased (egg) predation pressure by flatworms under ALAN can potentially strongly impact population dynamics of *Daphnia,* given that typhoplanid flatworms can be important predators of *Daphnia* [19]. This may not only affect *Daphnia* densities and top-down control of phytoplankton but may also lead to profound changes in zooplankton community composition (e.g. [31]). This is because *S. simplex* is a parasite of eggs that are in the brood chamber of *Daphnia*, which likely makes large-bodied individuals and species more vulnerable than smaller ones (as shown for copepods predating on *Daphnia* eggs [32]). For example, increased fish predation on zooplankton under ALAN reduced the mean body size of zooplankton and changed the zooplankton community structure [33]. It remains to be tested whether ALAN has similar effects on the flatworm–*Daphnia* interaction, potentially shaping zooplankton population structure, community composition, and top-down control of phytoplankton.

The effects of ALAN on species interactions and ecosystem functions may be further exacerbated by other stressors, a key one in an urbanization and climate change context being warmer night temperatures [34]. A recent study revealed that higher temperatures increased the top-down control of predatory *Mesostoma* flatworms on *Daphnia*, changing the zooplankton community structure and affecting algal biomass [31]. Therefore, we suggest further exploration of the interaction between warming, ALAN and flatworm parasitism on *Daphnia* population dynamics covering time periods that exceed the one in the present study, in urban ponds that are typically exposed to both the heat island effect and the light pollution.

## Data Availability

Data from the experimental trial are available from the Figshare repository [35]. Supplementary material is available online [36].

## References

[1] Guiden PW, Bartel SL, Byer NW, Shipley AA, Orrock JL. 2019 Predator–prey interactions in the Anthropocene: reconciling multiple aspects of novelty. Trends Ecol. Evol. **34**, 616–627. (10.1016/j.tree.2019.02.017) 30902358

[2] Gaston KJ, Visser ME, Hölker F. 2015 The biological impacts of artificial light at night: the research challenge. Phil. Trans. R. Soc. B **370**, 20140133. (10.1098/rstb.2014.0133) 25780244 PMC4375372

[3] Sanders D, Frago E, Kehoe R, Patterson C, Gaston KJ. 2021 A meta-analysis of biological impacts of artificial light at night. Nat. Ecol. Evol. **5**, 74–81. (10.1038/s41559-020-01322-x) 33139919

[4] Hölker F *et al*. 2021 11 Pressing research questions on how light pollution affects biodiversity. Front. Ecol. Evol. **9**, 896. (10.3389/fevo.2021.767177)

[5] Seymoure B, Dell A, Hölker F, Kalinkat G. 2023 A framework for untangling the consequences of artificial light at night on species interactions. Phil. Trans. R. Soc. B **378**, 20220356. (10.1098/rstb.2022.0356) 37899016 PMC10613547

[6] Oertli B, Parris KM. 2019 Review: toward management of urban ponds for freshwater biodiversity. Ecosphere **10**, e02810. (10.1002/ecs2.2810)

[7] Hölker F, Jechow A, Schroer S, Tockner K, Gessner MO. 2023 Light pollution of freshwater ecosystems: principles, ecological impacts and remedies. Phil. Trans. R. Soc. B **378**, 20220360. (10.1098/rstb.2022.0360) 37899012 PMC10613548

[8] Kühne JL, van Grunsven RHA, Jechow A, Hölker F. 2021 Impact of different wavelengths of artificial light at night on phototaxis in aquatic insects. Integr. Comp. Biol. **61**, 1182–1190. (10.1093/icb/icab149) 34180520

[9] Marangoni LFB *et al*. 2022 Impacts of artificial light at night in marine ecosystems—a review. Glob. Chang. Biol. **28**, 5346–5367. (10.1111/gcb.16264) 35583661 PMC9540822

[10] Ganguly A, Candolin U. 2023 Impact of light pollution on aquatic invertebrates: behavioral responses and ecological consequences. Behav. Ecol. Sociobiol. **77** 1–15. (10.1007/s00265-023-03381-z)

[11] Last KS, Hobbs L, Berge J, Brierley AS, Cottier F. 2016 Moonlight drives ocean-scale mass vertical migration of zooplankton during the arctic winter. Curr. Biol. **26**, 244–251. (10.1016/j.cub.2015.11.038) 26774785

[12] Moore MV, Pierce SM, Walsh HM, Kvalvik SK, Lim JD. 2000 Urban light pollution alters the diel vertical migration of Daphnia. Verh. Int. Verein. Limnol. **27**, 779–782. (10.1080/03680770.1998.11901341)

[13] Ludvigsen M *et al*. 2018 Use of an autonomous surface vehicle reveals small-scale diel vertical migrations of zooplankton and susceptibility to light pollution under low solar irradiance. Sci. Adv. **4**, eaap9887. (10.1126/sciadv.aap9887) 29326985 PMC5762190

[14] Maszczyk P, Tałanda J, Babkiewicz E, Leniowski K, Urban P. 2021 Daphnia depth selection in gradients of light intensity from different artificial sources: an evolutionary trap? Limnol. Oceanogr. **66**, 1367–1380. (10.1002/lno.11691)

[15] De Meester L, Mehner T, Scofield A. 2022 Diel vertical migration. In Encyclopedia of inland waters (eds T Mehner, K Tockner), pp. 281–291, 2nd edn. Oxford, UK: Elsevier. (10.1016/B978-0-12-819166-8.00166-3)

[16] Grubisic M *et al*. 2019 Light pollution, circadian photoreception, and melatonin in vertebrates. Sustainability **11**, 6400. (10.3390/su11226400)

[17] Tüzün N, Lemke N, Diez YL, Artois T, Monnens M. 2025 Tiny killers: first record of rhabdocoel flatworms feeding on water flea embryos. Ecol. Evol. **15**, e71277. (10.1002/ece3.71277) 40416756 PMC12100763

[18] De Meester L, Dumont HJ. 1990 Laboratory observations on the vertical distribution of a tropical pelagic flatworm (Mesostoma sp.) in relation to satiation. Hydrobiologia **198**, 103–106. (10.1007/BF00048626)

[19] Dumont HJ, Rietzler AC, Han BP. 2014 A review of typhloplanid flatworm ecology, with emphasis on pelagic species. Inland Waters **4**, 257–270. (10.5268/IW-4.3.558)

[20] Hänel A *et al*. 2018 Measuring night sky brightness: methods and challenges. J. Quant. Spectrosc. Radiat. Transf. **205**, 278–290. (10.1016/j.jqsrt.2017.09.008)

[21] Jónasson PM. 1979 The Lake Mývatn ecosystem, Iceland. Oikos **32**, 289.

[22] Lenth RV *et al*. 2023 emmeans: Estimated Marginal Means, aka Least-Squares Means. See https://CRAN.R-project.org/package=emmeans.

[23] Pinheiro J, Bates D, R.Core Team. 2023 nlme: Linear and Nonlinear Mixed Effects Models. R package version 3.1-163. See https://CRAN.R-project.org/package=nlme.

[24] R Core Team. 2023 R: a language and environment for statistical computing. R foundation for statistical computing. Vienna, Austria. See https://ropensci.org/blog/2021/11/16/how-to-cite-r-and-r-packages/.

[25] Hirt MR, Evans DM, Miller CR, Ryser R. 2023 Light pollution in complex ecological systems. Phil. Trans. R. Soc. B **378**, 20220351. (10.1098/rstb.2022.0351) 37899008 PMC10613538

[26] Poulin R. 2023 Light pollution may alter host–parasite interactions in aquatic ecosystems. Trends Parasitol. **39**, 1050–1059. (10.1016/j.pt.2023.08.013) 37722935

[27] Decaestecker E, De Meester L, Ebert D. 2002 In deep trouble: habitat selection constrained by multiple enemies in zooplankton. Proc. Natl Acad. Sci. USA **99**, 5481–5485. (10.1073/pnas.082543099) 11960005 PMC122795

[28] Diamantopoulou C, Christoforou E, Dominoni DM, Kaiserli E, Czyzewski J, Mirzai N, Spatharis S. 2021 Wavelength-dependent effects of artificial light at night on phytoplankton growth and community structure. Proc. R. Soc. B **288**, 20210525. (10.1098/rspb.2021.0525) PMC8220282 34157871

[29] Li D, Huang J, Zhou Q, Gu L, Sun Y, Zhang L, Yang Z. 2022 Artificial light pollution with different wavelengths at night interferes with development, reproduction, and antipredator defenses of Daphnia magna. Environ. Sci. Technol. **56**, 1702–1712. (10.1021/acs.est.1c06286) 35014268

[30] Kupprat F, Hölker F, Kloas W. 2020 Can skyglow reduce nocturnal melatonin concentrations in Eurasian perch? Environ. Pollut. **262**, 114324. (10.1016/j.envpol.2020.114324) 32179225

[31] Devkota N, Salis RK, Hansson L. 2023 Warming reshapes the invertebrate predation pressure on the plankton community. Freshw. Biol. **68**, 365–377. (10.1111/fwb.14031)

[32] Gliwicz ZM, Lampert W. 1994 Clutch-size variability in Daphnia: body-size related effects of egg predation by cyclopoid copepods. Limnol. Oceanogr. **39**, 479–485. (10.4319/lo.1994.39.3.0479)

[33] Tałanda J, Maszczyk P, Babkiewicz E, Rutkowska K, Ślusarczyk M. 2022 The short-term effects of planktivorous fish foraging in the presence of artificial light at night on lake zooplankton. J. Plankton Res. (ed. M Koski), **44**, 942–946. (10.1093/plankt/fbac046) 36447780 PMC9692195

[34] Tougeron K, Sanders D. 2023 Combined light pollution and night warming as a novel threat to ecosystems. Trends Ecol. Evol. **38**, 701–704. (10.1016/j.tree.2023.05.012) 37286418

[35] Tüzün N, Hölker F, De Meester L. 2025 Data for flatworm–Daphnia trial under light pollution. Figshare. (10.6084/m9.figshare.28457171)

[36] Tüzün N, Hölker F, De Meester L. 2025 Supplementary material from: Artificial light at night intensifies effects of a parasitic flatworm on the water flea Daphnia magna. Figshare. (10.6084/m9.figshare.c.8007250)40987336

